# Mental health interventions targeting children and young people: A mapping review of interventions, follow-up, and evidence gaps

**DOI:** 10.12688/f1000research.123561.2

**Published:** 2025-05-08

**Authors:** Astrid Dahlgren, Ingrid Borren, Brynhildur Axelsdottir, Mari Elvsåshagen, Karianne Hammerstrøm Nilsen

**Affiliations:** 1 OsloMet, Faculty of Health Sciences, Oslo Metropolitan University, P.O. Box 4, St. Olavs plass, NO-0130 Oslo, Norway; 2 RBUP East and South, P.O. Box 4623 Nydalen, 0405 Oslo, Norway

**Keywords:** mental health, children, youth, interventions, mapping review, evidence gaps

## Abstract

**Background:**

Young people with mental disorders should be offered evidence-based treatments. In 2018, we developed a national evidence portal in Norway providing access to living evidence summaries. This work has been an important contribution to mental health care in Norway but is also a rich data source for exploring the existing research. At the time of this study, eight overviews of overviews (OoO) were published addressing the effects of treatments for attention deficit / hyperactivity disorder (ADHD), anxiety, depression, bipolar disorder, psychosis, obsessive compulsive disorder, self-harm and trauma / post-traumatic stress disorder. The objective of this study was to do a secondary analysis of this evidence to describe the state-of-the art in this field, and to map:

1. treatments evaluated for each diagnostic group and the longest time of follow-up
2. treatment comparisons evaluated for more than one diagnostic group

**Methods:**

We performed a mapping review. Data extraction was performed independently by two authors. All data was entered into Excel. Unique treatment comparisons were visualised in Sunburst diagrams. The longest time of follow-up and treatments evaluated across diagnostic groups were reported in descriptive tables.

**Results:**

We identified 200 treatment comparisons including a wide variety of interventions. Some diagnoses are treated mostly with pharmaceutical therapies (such as bipolar disorder) or psychological therapy alone or in combination (such as anxiety and psychosis). Others are treated with a broader spectrum of therapeutic approaches (such as depression and ADHD). The evidence supporting most treatments is of low to very low certainty. Ten percent of the comparisons included follow-up assessments beyond 12 months. Cognitive behavioural therapy, dialectic behavioural therapy, physical activity and mindfulness interventions were effective across populations.

**Conclusions:**

The evidence supporting treatment of mental disorders in young people has important limitations. Future research efforts should address these evidence gaps.

## Introduction

Acting early by providing children and youth with evidence-based treatments for mental health problems may prevent chronic illness and deterioration of other health outcomes and quality of life. ^1^ Attention-deficit / hyperactivity disorder (ADHD), behavioural disorders and internalising disorders such as anxiety and depression are common disorders diagnosed in childhood and adolescence. ^2^ While the etiology of these disorders may differ, they share commonalities, such as causing distress or impairment in key areas of functioning. In general, as many as one in four are reported to experience a mental disorder, and according to the WHO mental health disorders are among the leading causes of disability worldwide. ^1^^,^^2^

Interventions targeting mental disorders encompass a range of treatments including (but not restricted to) psychological therapies, pharmacological therapies, peer-supported interventions, educational interventions, physical activity, nutritional interventions and alternative therapies such as acupuncture and yoga. Traditionally, treatments for mental health problems have taken a diagnostic approach, however a new school of thought argue for a transdiagnostic approach that cuts across traditional diagnostic boundaries. ^3^

Children and young people should be offered evidence-based treatments. Summarised evidence is a necessity for good quality health care and should inform decision-making about treatment choices. ^4^^,^^5^ However, there is an abundance of systematic reviews published every year and many of these are not of satisfactory quality. ^6^ Evidence summaries such as overviews of overviews are therefore important decision-making tools for users, professionals and policy makers. ^7^

In 2018 we developed a national evidence portal (see here) in Norway hosting living evidence summaries of systematic reviews evaluating the effects of interventions for children and young people with mental disorders. ^8^^,^^9^ Living evidence summaries are systematic reviews being continuously updated and revised, using a systematic approach to literature search, screening of new publications for possible inclusion, data extraction and GRADE-assessments.

The evidence portal relies on best practice review methodology and was inspired by other international evidence portals targeting mainly adults and somatic illness. ^10^ At the time the present study was conducted, eight overviews of overviews (here termed ‘OoOs’) had undergone peer-review and were published in the evidence portal. In an OoO, all available systematic reviews meeting explicit inclusion and exclusion criteria are summarised and quality assessed. Sometimes OoOs are also called umbrella reviews. Each of the OoOs addressed a specific diagnostic group and summarised the effects of treatments for children and young people with ADHD, anxiety, depression, bipolar disorder, psychosis (including schizophrenia), obsessive-compulsive disorder (OCD) self-harm and trauma (including post-traumatic stress disorder, PTSD) respectively. ^11^^–^^18^

This immense work has been an important contribution to mental health care in Norway and has been integrated in the Norwegian national guidelines but also provides an opportunity for exploring the characteristics and evidence gaps of the existing research. This led us to conduct the study reported on in this paper, were we wanted to explore the diversity of treatments evaluated and to map their effects across diagnostic groups.

This is valuable knowledge for researchers planning new treatment evaluations for children and young people, but also for providing input to the debate on transdiagnostic approaches, and the potential value of some treatments to be effective for several mental disorders. ^3^

## Objectives

The purpose of this study was to do a secondary analysis of the eight evidence summaries included in the evidence portal and to describe the state of the art of the existing research in this field. Specifically, we aimed to:

(1) map all treatments evaluated for each diagnostic group and the longest time of follow-up included in these assessments
(2) map treatment comparisons evaluated across groups and to create an overview of the effects of these including the certainty of the evidence (using the *Grading* of Recommendations Assessment, Development and Evaluation framework – *GRADE -* on the primary outcome for each diagnostic group

To our knowledge, no mapping review exploring these aims have previously been conducted.

## Methods

### Design

This study is a mapping review and a secondary analysis of eight overviews of overviews (OoO). There is no gold standard for conducting mapping reviews, however a common set of guiding rules have been suggested. ^19^^,^^20^ In general, mapping reviews provide an overview of the literature by describing and organising the research literature according to characteristics such as interventions, intervention components or population. Mapping reviews are also used for identifying evidence gaps, and to inspire and guide new research initiatives. ^19^^,^^20^ The protocol for this mapping review has been published. ^21^

### Description of the included overviews of systematic reviews

There is an abundance of systematic reviews summarising the effects of interventions for child and young people’s mental health. We have in the above-mentioned previous work completed eight OoOs including systematic reviews evaluating all interventions targeting children and adolescents (0-18 years) with; ADHD, anxiety, depression, bipolar disorder, psychosis (including schizophrenia), OCD, self-harm and trauma (including PTSD). ^11^^–^^18^

The analysis reported on in this paper is in its entirety based on these eight OoOs and represents a secondary analysis of what we considered at the time of the publication of this manuscript to be complete summaries of the effectiveness of interventions reported on in high quality systematic reviews.

The protocols for the included overviews have been published in PROSPERO (ADHD, CRD42020159885; Anxiety, CRD42020159884; Bipolar, CRD42020176356; Depression, CRD42020159883; Psychosis, CRD42020212244; Self-harm, CRD42019117942; Trauma CRD42019120078; OCD, CRD42020221081), however we briefly summarise the methods used here.

**Figure 1.**
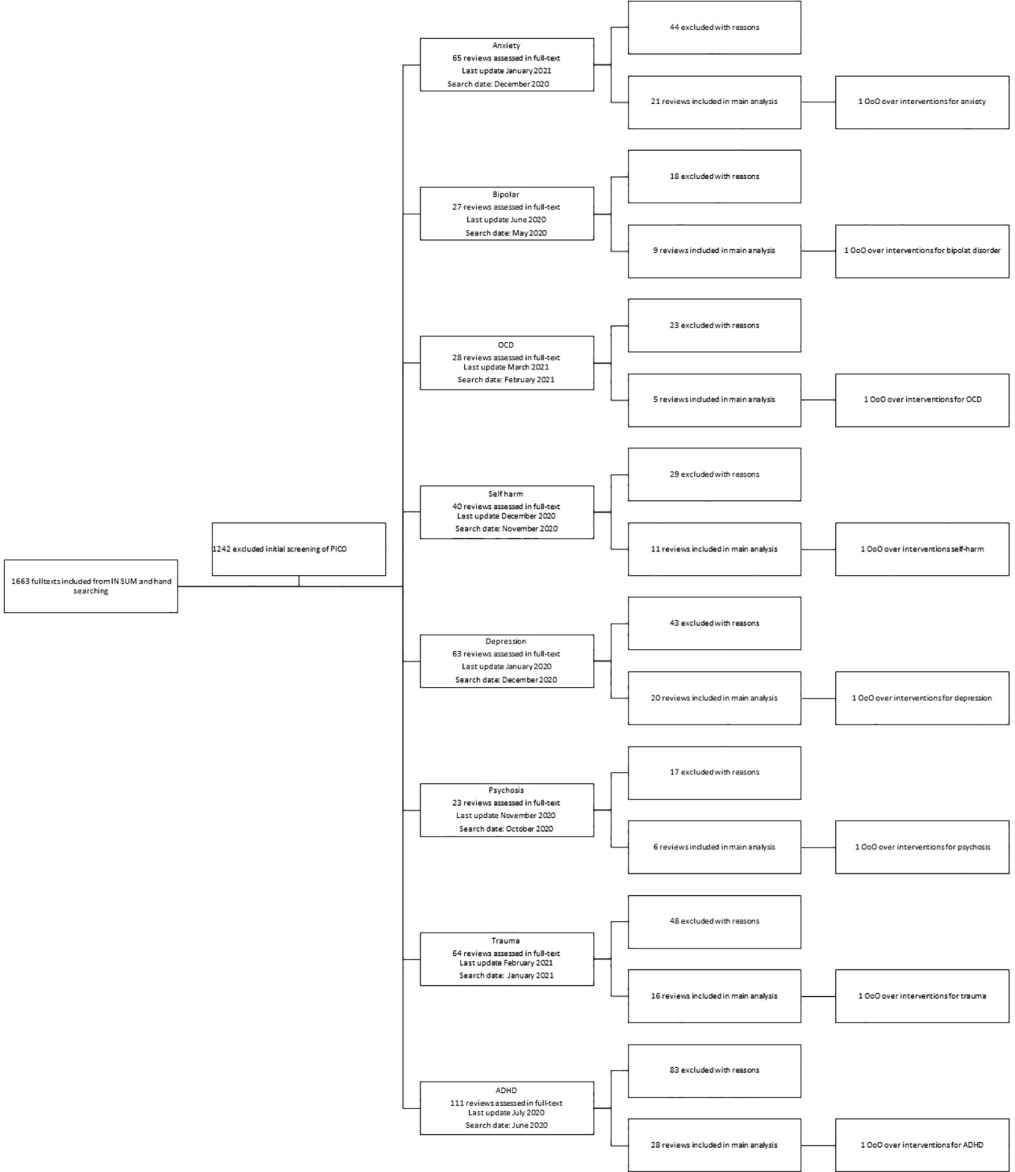
Preferred Reporting Items for Systematic Reviews and Meta-Analyses (PRISMA) flowchart. PICO, population intervention comparison outcome; OCD, obsessive compulsive disorder; ADHD, attention deficit / hyperactivity disorder; OoO, overviews of overviews.

All OoOs were conducted adhering to the Preferred Reporting Items for Systematic Reviews and Meta-Analyses (PRISMA) checklist and according to “best practice”-principles of review methods. ^10^ The search strategies for the OoOs were largely based on the database for systematic reviews IN SUM (indexing publications from all major databases. The search strategy applied by the IN SUM database can be accessed here). All systematic reviews indexed in IN SUM were considered for inclusion. We also hand searched national databases of evidence-based guidelines in Sweden, Denmark and the UK (NICE). The flow chart describing each step of the inclusion and exclusion of publications can be seen in Figure 1.

To be included in one of the OoOs, a review had to meet a set of minimum criteria (4 out of 5) for being considered “systematic”, set by DARE. ^22^

1. Clear inclusion and exclusion criteria
2. A comprehensive search strategy
3. A compilation of results from included studies, or
4. Quality assessment of included studies.
5. Sufficiently reported details about the individual included studies

Furthermore, all interventions intended to improve an aspect of mental health for children and adolescents under the age of 18 were included.

### Data extraction and evidence included in the analysis of the mapping review

We mapped all unique interventions evaluated in the OoOs and sorted them by the following categories: medication therapies, combination therapies, training interventions (such as skills, training and psychoeducation), dietary interventions, psychosocial therapies, systemic interventions, physical interventions and other interventions. In the results section, we present these intervention categories in descriptive charts (Sunburst-diagrams) for each diagnostic group respectively. We also mapped the number of treatment comparisons evaluated and the longest time of follow-up included in these assessments (for any outcome).

To create a mega-map of intervention effects across diagnostic groups we also extracted data on the primary outcome for each intervention, and the judgement of certainty for this outcome. ^23^^,^^24^ All data were entered into Excel. The primary outcome was conceptualised as overall symptomatology associated with the relevant condition, such as anxiety symptoms for those with anxiety and depression symptoms for those with depression. In cases where the same outcome was assessed using different outcome measures, we extracted the outcome with the longest follow up and highest certainty. ^23^ If a review did not report findings on overall symptoms, we reported other symptoms as proxy, for example inattention in treatment of ADHD when total symptoms were not available.

Since this is a mapping review, we based our analysis on the effect-sizes and judgements of certainty as they were reported in the OoO. Judgements about certainty included in the OoO were made using the GRADE-criteria. ^23^^,^^24^ GRADE (Grading of Recommendations, Assessment, Development and Evaluations) is a transparent and widely adapted tool for developing and presenting summaries of evidence. ^23^ Using this framework, the evidence is judged to have high, moderate, low or very low certainty by considering the following criteria: risk of bias, imprecision, inconsistency, indirectness, publication bias, magnitude of effect, dose-response gradient and residual confounding.

We extracted the effect sizes and categorized these “small”, “moderate” or “large” based on statistical rule-of-thumb judgements (see Table 1). ^10^^,^^24^ For outcomes judged to be of very low certainty, effect sizes were reported as “?” adhering to the GRADE- recommendations. ^23^

**Table 1.** Rule of thumb thresholds used for effect sizes.

Effect estimate	Small effect	Moderate effect	Large effect
Relative risk (RR)			RR >2
Odds ratio (OR)	1.68	3.47	6.71
Standardised mean difference (SMD), Cohen’s d and Hedge’s g	<0.2	0.5	>0.8
Number needed to treat (NNT)	>10	2-10	1
Remission	20%	50%	100%

What is considered “a meaningful effect” depends on the individual person and context. Thus, we made no attempt to consider the clinical importance of these results as this is best judged by those delivering and receiving treatments. In some cases, it was difficult to judge the size of the effect as the results were not reported using standardised or relative effect estimates. In these circumstances we used the systematic review authors’ own judgements and have annotated these in the results table. If no judgements were made by the review authors, we marked this as “effective” without making judgements about the size of effect. Trivial or small not statistically significant differences in effect was coded as “little or no difference” according to the GRADE-recommendations. ^25^

All data extraction was done by one author and double checked by another co-author. Any difference in opinion between these were discussed with a third co-author.

The OoOs are “living reviews”, and so for the sake of this secondary analysis we based our data collection on the latest updated version of each overview respectively, with the most recent of these updates including evidence published in April 2021 or later. The data collection for this mapping review took place between September 2021 and December 2022. On average, the OoO were updated on a yearly basis at the time of this publication.

### Evidence not included in the mapping review

Some of the OoOs included both preventive and treatment interventions. For this mapping review, we only considered interventions targeting children and adolescents with a clinical diagnosis of mental disorder or otherwise judged by the systematic review authors to have moderate to serious symptomatology. The reason for this was that not all the OoOs included preventive studies and thus would render this secondary analysis incomplete.

The OoOs did not include full data extraction and GRADE-assessments of studies where pharmacological interventions were not compared to active interventions, thus this secondary analysis does not include such comparisons. Furthermore, studies evaluating mental health interventions targeting people where the primary health concern was somatic (e.g., people with asthma and co-occurring anxiety) were excluded in the OoOs and consequently not included in the secondary analysis.

## Results

### Treatment comparisons and follow-up

We reviewed 116 systematic reviews included in the eight OoOs (see Figure 1). As the OoOs are living documents and regularly updated, we have included a list of the systematic reviews included in the summaries at the time data was extracted for this study – see *Underlying data.* ^30^

The evidence underlying treatment options of mental disorders in children and young people includes 200 treatment comparisons evaluating the primary outcome for each specific diagnostic group. See *Underlying data* ^29^ for more information.

Overall, 49.5% of the treatment comparisons were non-pharmacological interventions, 36% included pharmacological or nutrition/supplemental interventions, and 14.5% were combination treatments including both pharmacological and non-pharmacological treatments.

The number of treatment comparisons varied greatly across the diagnostic groups; from 69 comparisons for ADHD to 7 for OCD (see Table 2).

**Table 2.** Number of treatment comparisons by diagnostic group. ADHD, attention deficit / hyperactivity disorder; OCD, obsessive compulsive disorder; PTSD, post-traumatic stress disorder.

Diagnostic group	Treatment comparisons
ADHD	69
Anxiety	14
Bipolar	18
Depression	20
OCD	7
Psychosis	29
PTSD	16
Self-harm	14
Trauma	13
**Total**	**200**

### Interventions evaluated by diagnostic group

**Figure 2.**
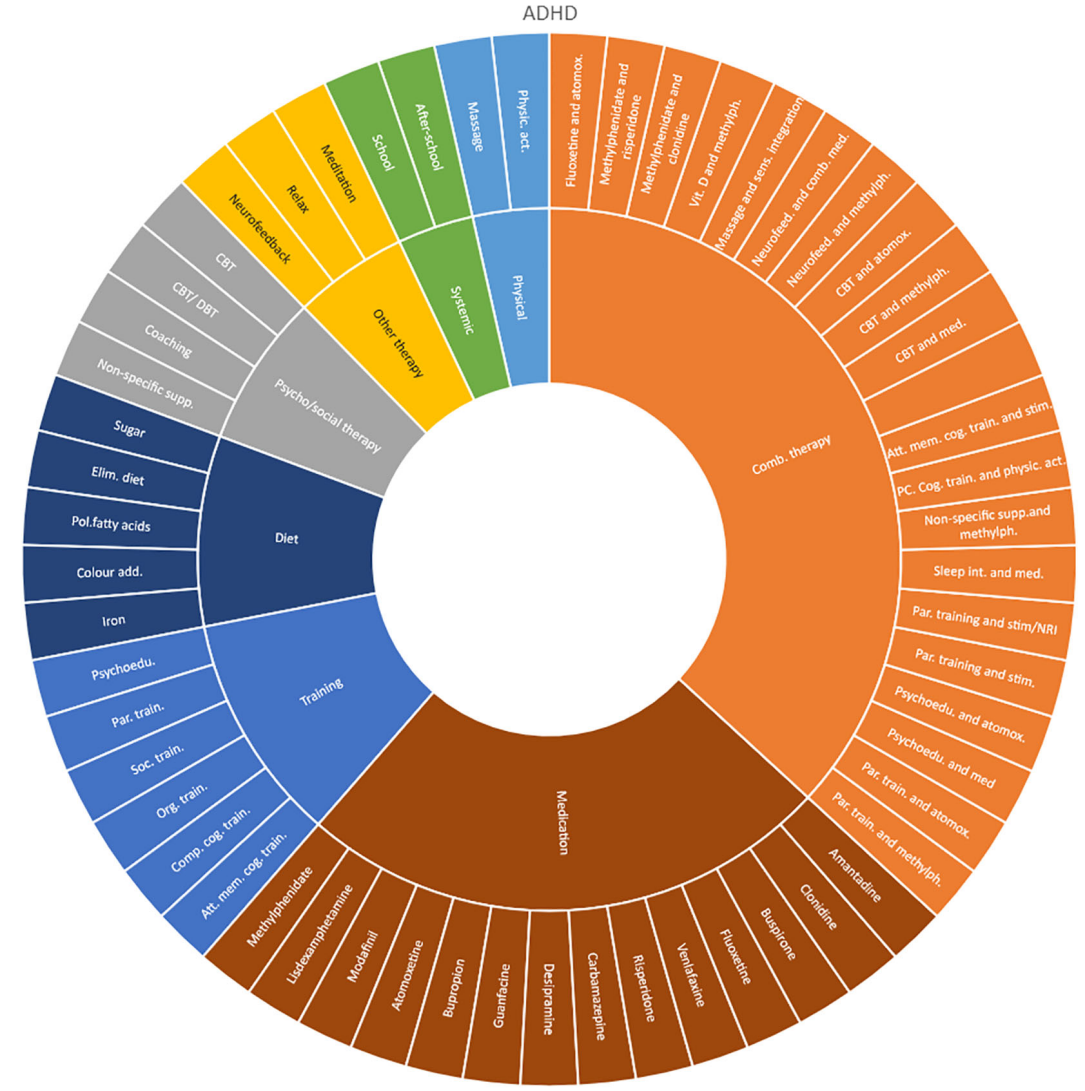
Unique intervention categories evaluated for attention deficit / hyperactivity disorder (ADHD). CBT, cognitive behavioural therapy; DBT, dialectic behavioural therapy; NRI, norepinephrine reuptake inhibitors.

**Figure 3.**
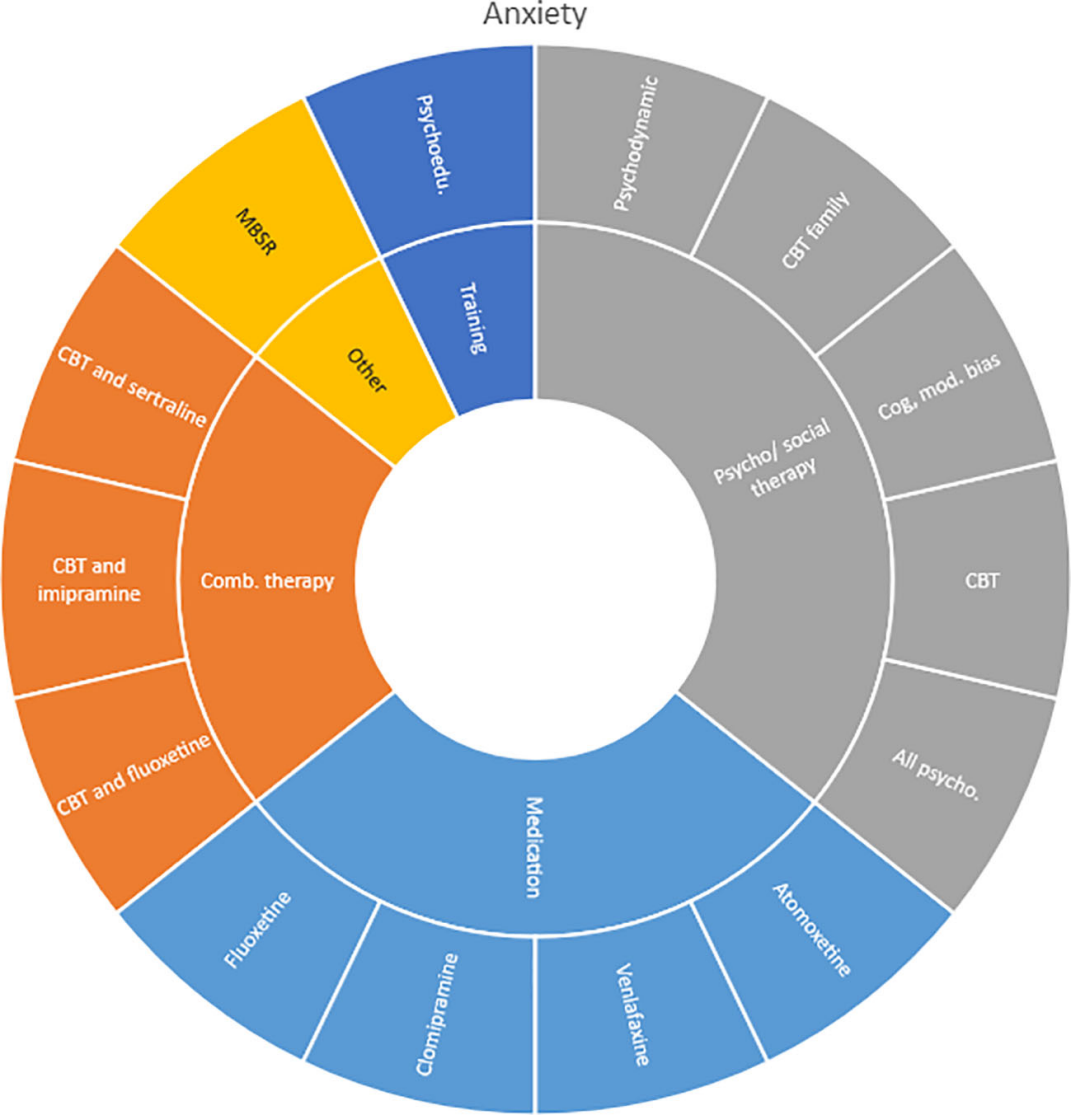
Unique intervention categories evaluated for anxiety. CBT, cognitive behavioural therapy; MBSR, mindfulness-based stress reduction.

**Figure 4.**
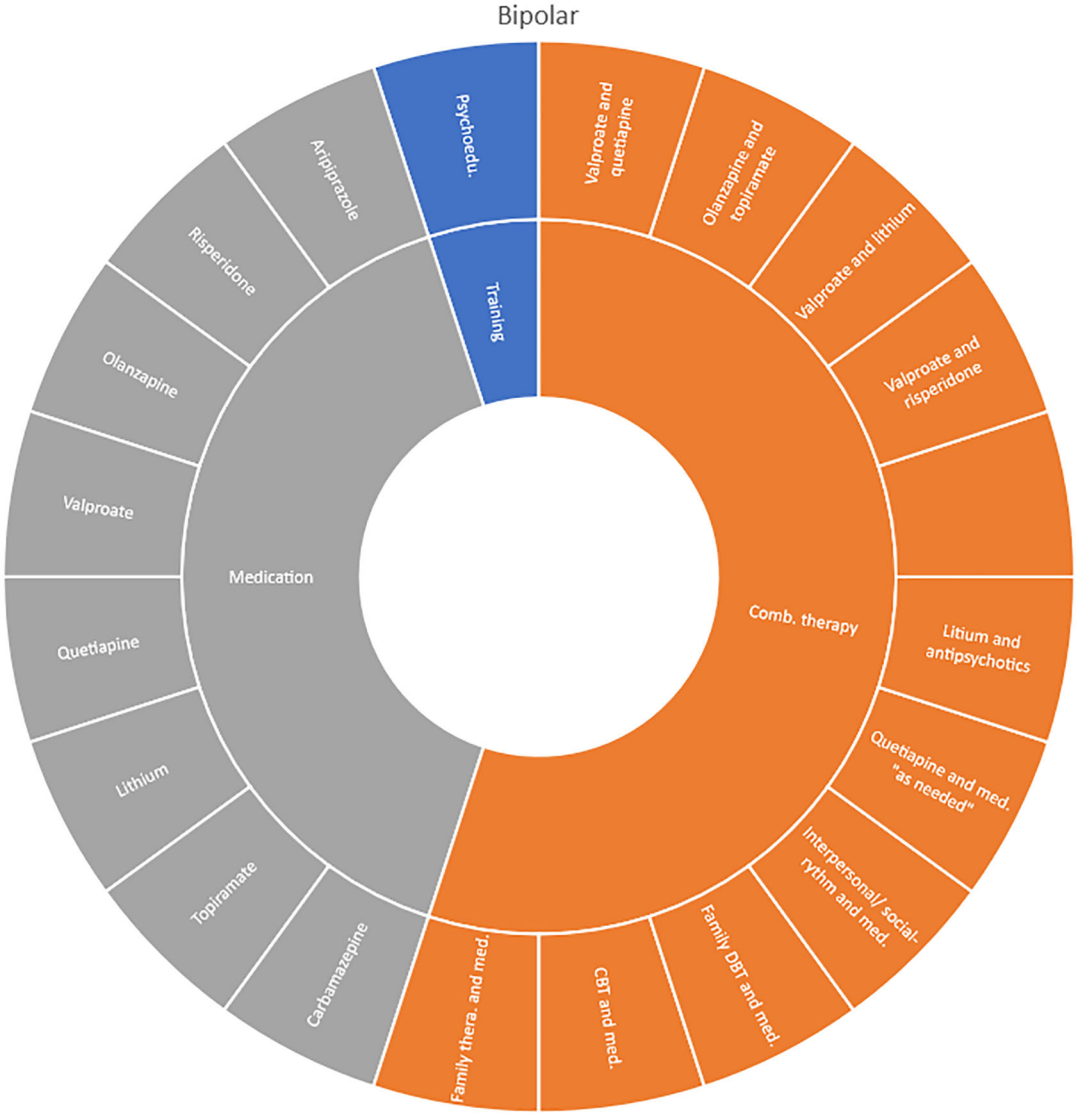
Unique intervention categories evaluated for bipolar disorder. CBT, cognitive behavioural therapy; DBT, dialectic behavioural therapy.

**Figure 5.**
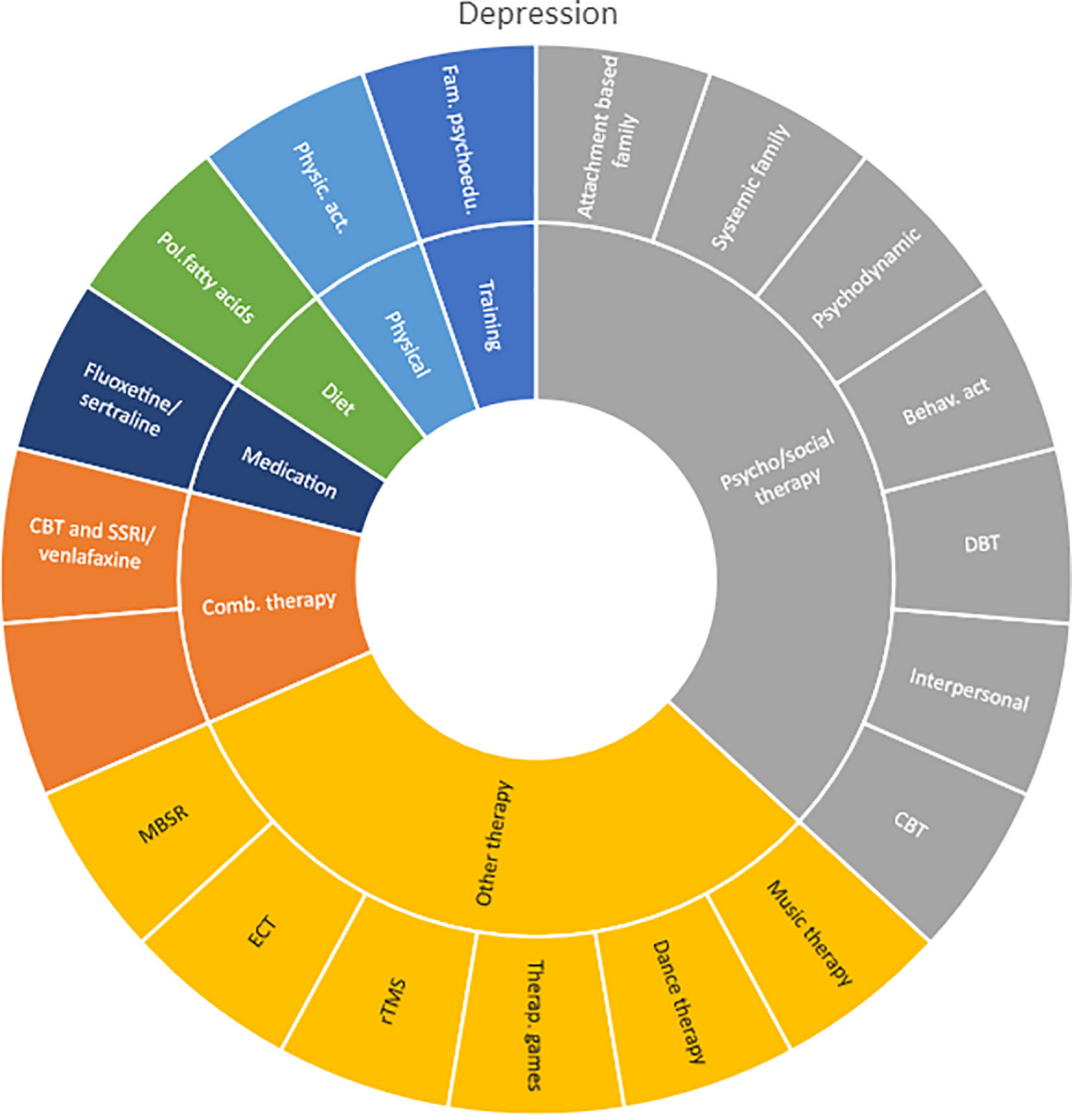
Unique intervention categories evaluated for depression. CBT, cognitive behavioural therapy; DBT, dialectic behavioural therapy; ECT, electroconvulsive therapy; rTMS, repetitive transcranial magnetic stimulation; MBSR, mindfulness-based stress reduction.

**Figure 6.**
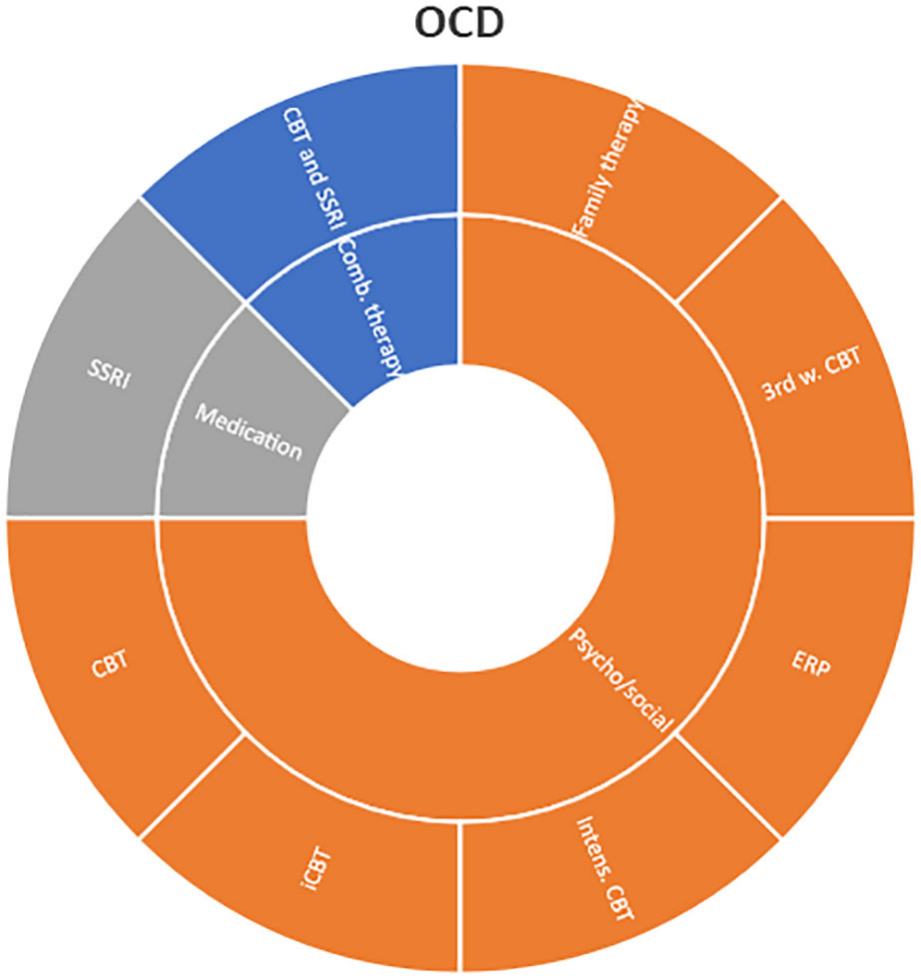
Unique intervention categories evaluated for obsessive compulsive disorder (OCD). CBT, cognitive behavioural therapy; iCBT, internet-based cognitive behavioural therapy; ERP, exposure and response prevention; SSRI, selective serotonin reuptake inhibitors.

**Figure 7.**
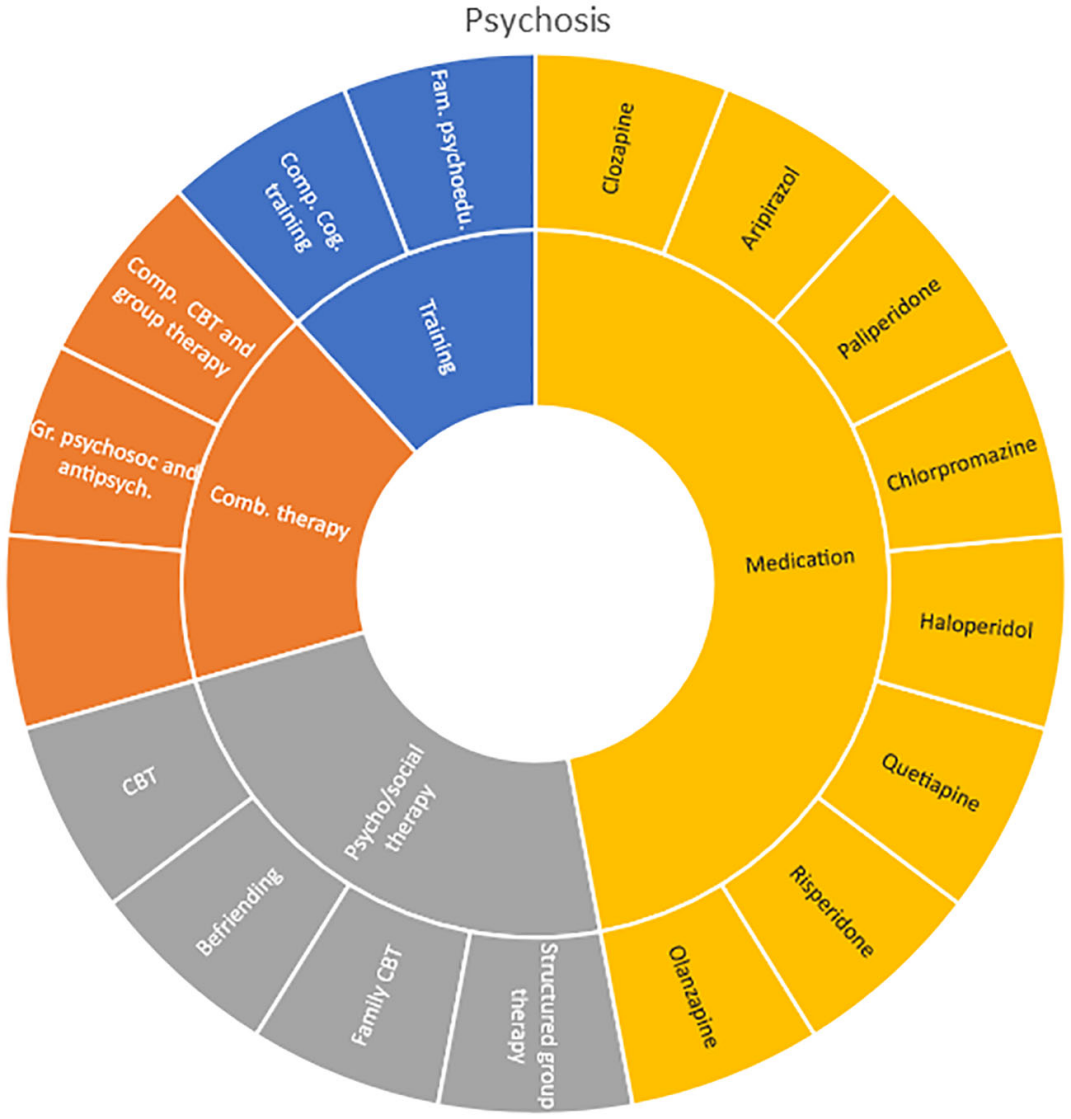
Unique intervention categories evaluated for psychosis. CBT, cognitive behavioural therapy.

**Figure 8.**
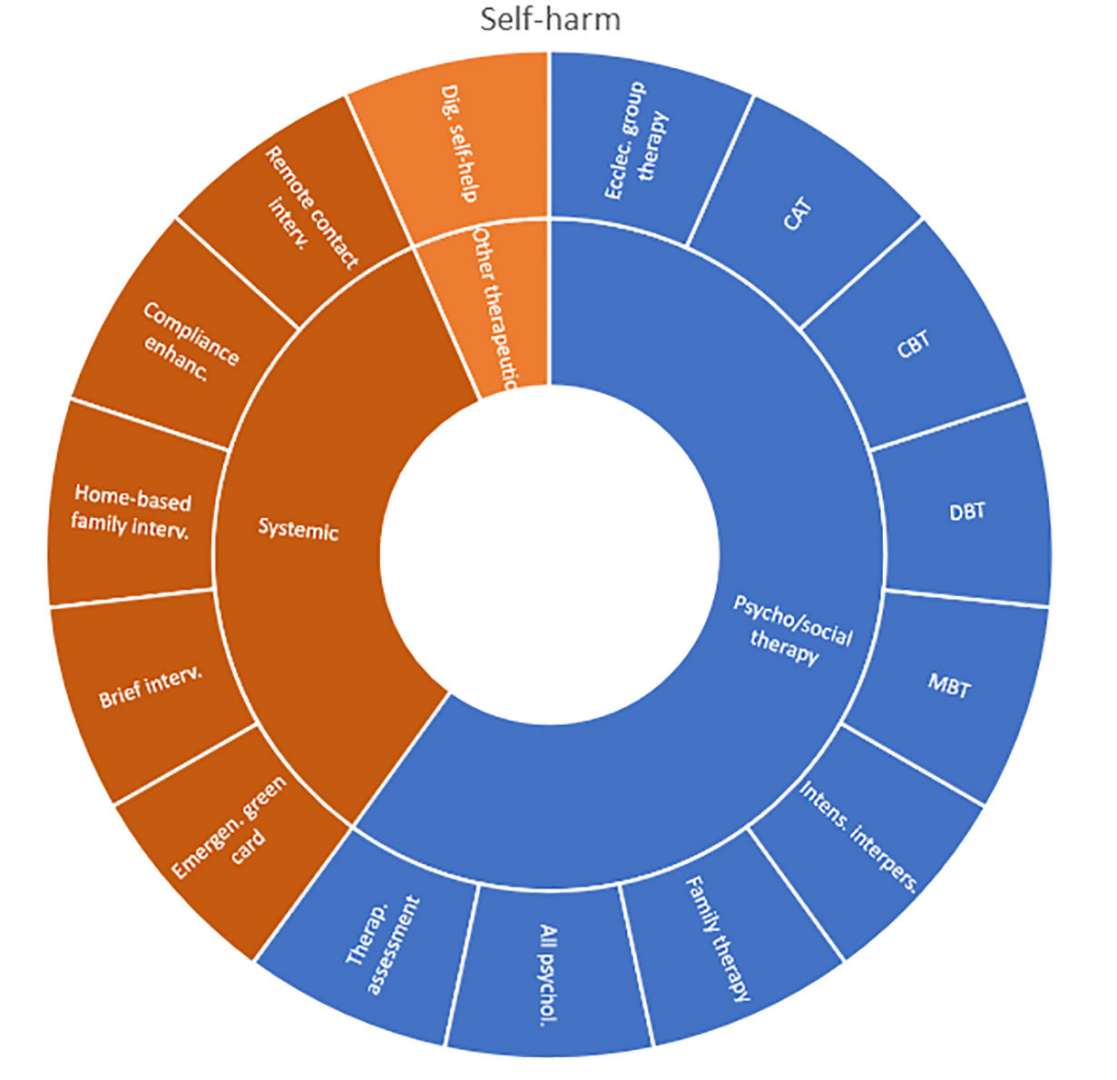
Unique intervention categories evaluated for self-harm. CAT, cognitive analytic therapy; CBT, cognitive behavioural therapy; DBT, dialectic behavioural therapy; MBT, mentalization-based therapy.

**Figure 9.**
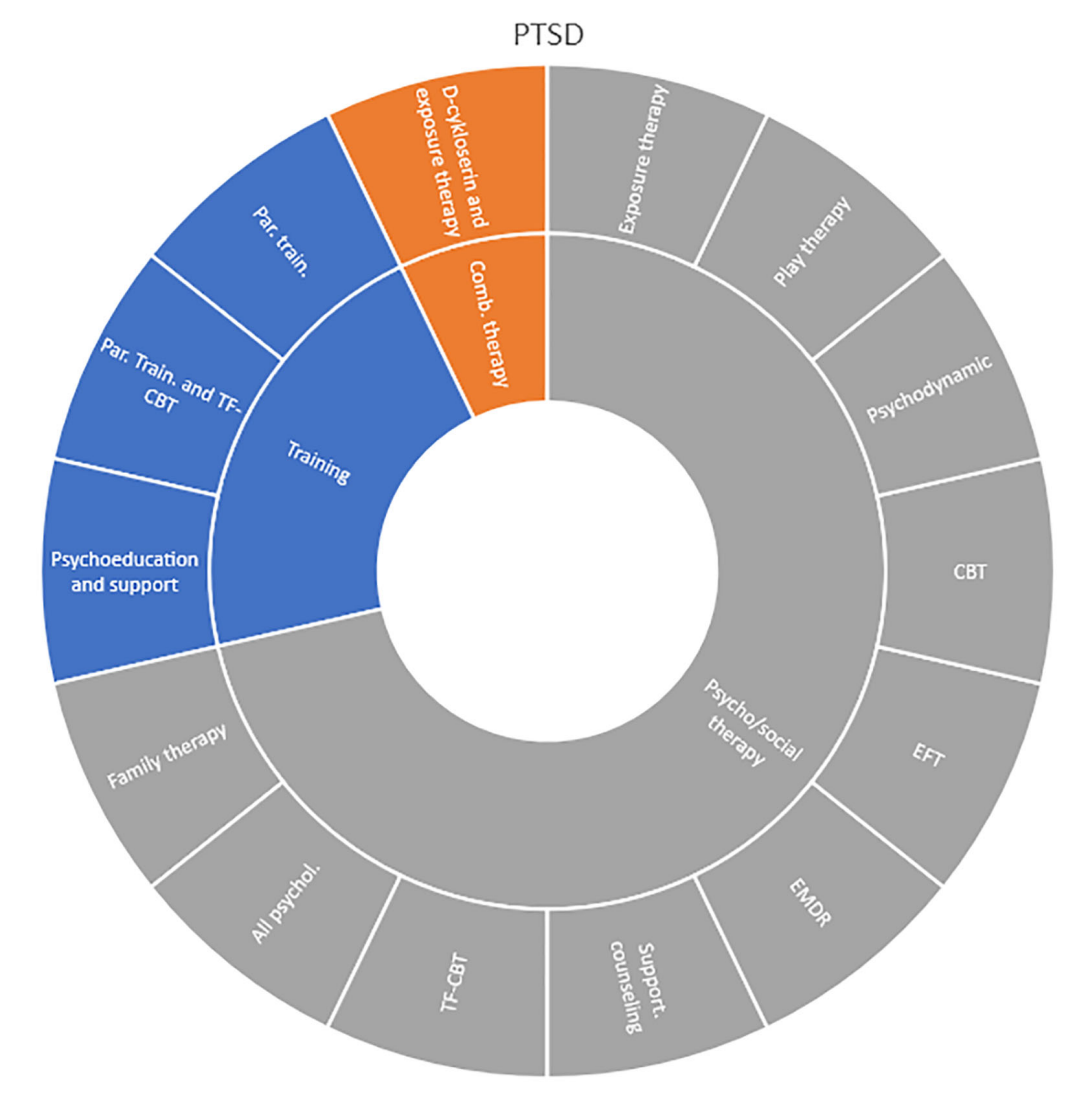
Unique intervention categories evaluated for post-traumatic stress disorder (PTSD). CBT, cognitive behavioural therapy; EFT, emotion-focused therapy; EMDR, eye movement desensitisation and reprocessing; TF-CBT, trauma focused cognitive behavioural therapy.

**Figure 10.**
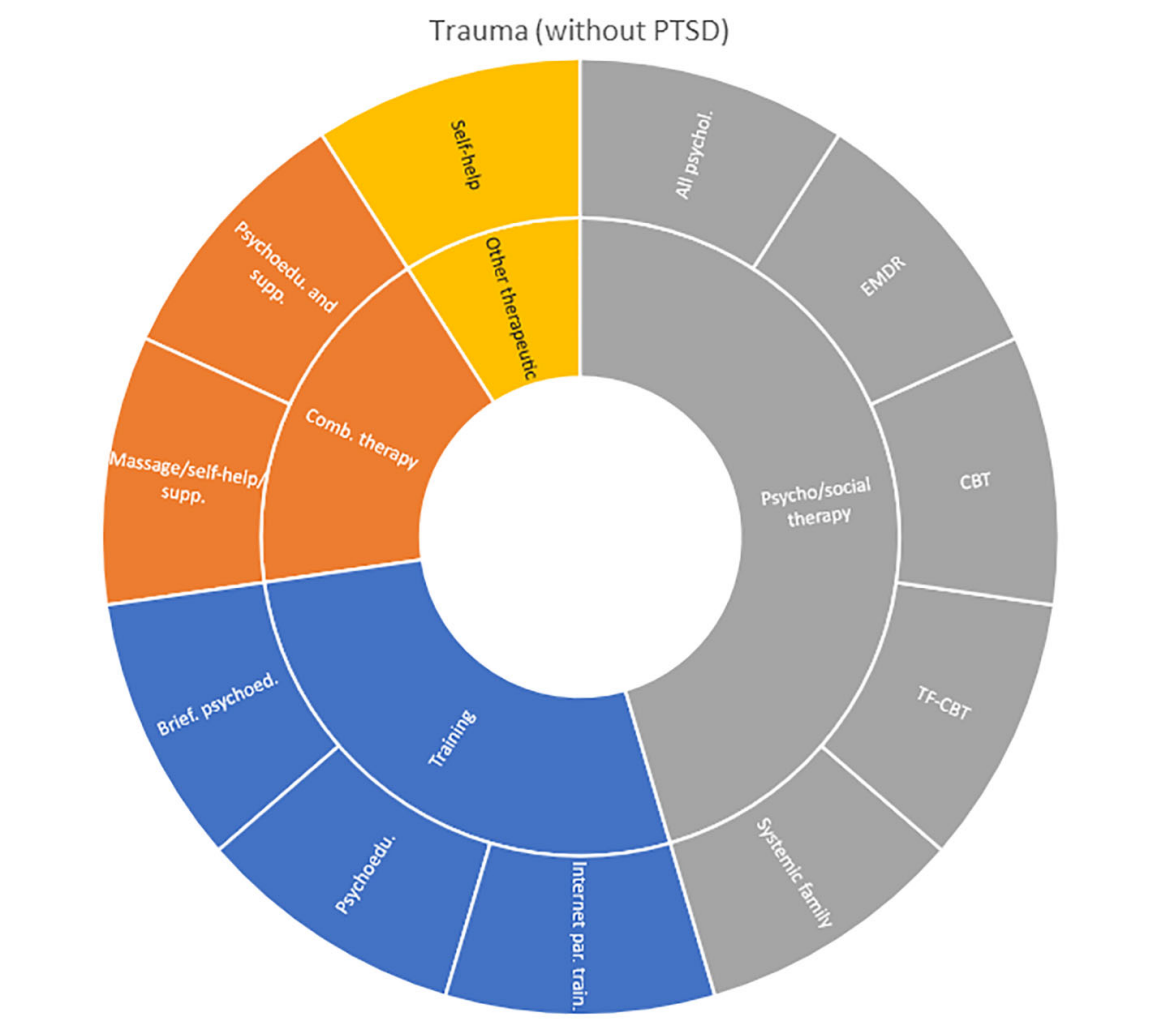
Unique intervention categories evaluated for trauma. EMDR, eye movement desensitisation and reprocessing; CBT, cognitive behavioural therapy; TF-CBT, trauma focused cognitive behavioural therapy.

Interventions by treatment categories are displayed in Sunburst-diagrams by diagnostic groups (see Figures 2– 10). The evidence informing ADHD-treatment includes a range of psychological, psychosocial, dietary, systemic, physical activity, skills training and pharmaceutical interventions. About half of the ADHD-interventions are pharmacological or combination therapies including medication.

Most treatment evaluations for depression are non-pharmacological and include different types of psychological therapies, physical activity, art therapy and medical treatments such as electroconvulsive therapy (ECT) and repetitive transcranial magnetic stimulation (rTMS). The evidence also includes pharmacological interventions including use of serotonin-norepinephrine reuptake inhibitors and selective serotonin reuptake inhibitors (SNRIs/SSRIs) in combination with cognitive behavioural therapy (CBT) or SSRI used alone.

The evidence for treating OCD represents less diversity, and most interventions are CBT-based, either used alone or in combination with medications.

Treatment evaluations for bipolar disorder and psychosis include mainly pharmacological treatments, either as direct comparisons or in combination with psychological treatments or other medications (bipolar disorder). Psychological treatments used for psychosis include psychoeducation, computer-based cognitive training, CBT and group- and family therapy. For bipolar disorder, evaluations also include psychoeducation, CBT, interpersonal therapy, Dialectic behavioural therapy (DBT) and family therapy.

Self-harm and trauma/PTSD-interventions are all psychological, psychosocial, or other therapeutic interventions such as psychoeducation. Several organisational or systemic treatments used for preventing suicide or reoccurrence of self-harm have also been evaluated, including interventions such as the use of “emergency green card” (self-admittance to specialist health care).

### Time of follow-up

The longest follow-up for any outcome could be found for anxiety (6 years), followed by ADHD and depression (3 years), bipolar, self-harm and PTSD (2 years), trauma (1 year) and OCD (6 months).

Overall, approximately 40% of the treatment comparisons included only short-term follow-up (< 3 months). A total of 10% of the treatment comparisons included follow-up beyond 12 months (see Table 3).

**Table 3.** The longest follow-up times (by any outcome).

Time of follow up	%
End of treatment to 3 months	39.50%
3 to 6 months	16.00%
7 to 12 months	15.50%
13 to 18 months	3.50%
19 to 24 months	4.00%
25 months and more	2.50%
Unclear	19.00%
**Grand total**	**100.00%**

Follow-up times by diagnosis and category can be seen in Table 4. Overall, it can be observed that for most diagnostic groups, treatment comparisons of pharmacological treatment included very short follow-up.

**Table 4.** Follow-up times by diagnostic group and outcome category. ADHD, attention deficit / hyperactivity disorder; OCD, obsessive compulsive disorder; PTSD, post-traumatic stress disorder.

Follow up by diagnosis	%
ADHD	
Non-pharm (29% of comparisons)	
End of treatment to 3 months	10%
3 to 6 months	15%
7 to 12 months	30%
13 to 18 months	5%
19 to 24 months	10%
25 months and more	5%
Unclear	25%
Non-pharm/pharm (22% of comparisons)	
End of treatment to 3 months	33%
3 to 6 months	20%
7 to 12 months	20%
13 to 18 months	13%
Unclear	13%
Pharm/suppl (49% of comparisons)	
End of treatment to 3 months	59%
3 to 6 months	24%
7 to 12 months	3%
13 to 18 months	6%
Unclear	9%
Anxiety	
Non-pharm (57% of comparisons)	
End of treatment to 3 months	38%
3 to 6 months	13%
7 to 12 months	13%
Unclear	38%
Non-pharm/pharm (36% of comparisons)	
25 months and more	20%
Unclear	80%
Pharm/suppl (7% of comparisons)	
Unclear	100%
Bipolar	
Non-pharm (6% of comparisons)	
19 to 24 months	100%
Non-pharm/pharm (22% of comparisons)	
End of treatment to 3 months	75%
7 to 12 months	25%
Pharm/suppl (72% of comparisons)	
End of treatment to 3 months	62%
Unclear	38%
Depression	
Non-pharm (80% of comparisons)	
End of treatment to 3 months	38%
3 to 6 months	19%
7 to 12 months	6%
25 months and more	6%
Unclear	31%
Non-pharm/pharm (5% of comparisons)	
Unclear	100%
Pharm/suppl (15% of comparisons)	
3 to 6 months	33%
7 to 12 months	67%
OCD	
Non-pharm (71% of comparisons)	
End of treatment to 3 months	80%
3 to 6 months	20%
Non-pharm/pharm (29% of comparisons)	
End of treatment to 3 months	50%
Unclear	50%
Psychosis	
Non-pharm (24% of comparisons)	
3 to 6 months	14%
7 to 12 months	43%
19 to 24 months	14%
25 months and more	14%
Unclear	14%
Non-pharm/pharm (7% of comparisons)	
7 to 12 months	50%
Unclear	50%
Pharm/suppl (69% of comparisons)	
End of treatment to 3 months	75%
7 to 12 months	10%
19 to 24 months	5%
25 months and more	5%
Unclear	5%
PTSD	
Non-pharm (94% of comparisons)	
End of treatment to 3 months	33%
3 to 6 months	7%
7 to 12 months	40%
13 to 18 months	7%
19 to 24 months	13%
Pharm/suppl (6% of comparisons)	
End of treatment to 3 months	100%
Self-harm	
Non-pharm (100% of comparisons)	
End of treatment to 3 months	14%
3 to 6 months	21%
7 to 12 months	14%
13 to 18 months	7%
19 to 24 months	7%
Unclear	36%
Trauma	
Non-pharm (100% of comparisons)	
End of treatment to 3 months	31%
3 to 6 months	54%
7 to 12 months	15%

### Treatment uncertainty and comparative effectiveness

There was a large diversity of treatment comparisons evaluated. We identified 24 unique groups of treatment comparisons evaluated for more than one diagnostic group. These can be seen in Table 5. GRADE-judgements are colour-coded accordingly: green for moderate to high certainty, yellow is used for low certainty and red for very low certainty.

**Table 5.** Overview of shared treatment comparisons, intervention effects and certainty of the evidence. ADHD, attention deficit / hyperactivity disorder; OCD, obsessive compulsive disorder; PTSD, post-traumatic stress disorder; CBT, cognitive behavioural therapy; DBT, dialectic behavioural therapy; TAU, treatment as usual; TF-CBT, trauma focused cognitive behavioural therapy; EMDR, eye movement desensitisation and reprocessing.

	ADHD	Anxiety	Bipolar	Depression	OCD	PTSD	Trauma	Self-harm	Psychosis
Psychoeducation vs TAU	?/very low certainty		Effective/low certainty **	?/very low certainty			?/very low certainty		
Psychological vs TAU						Small effect/low certainty	Little or no difference/low certainty	Small effect/moderate certainty	
CBT vs TAU *	Moderate effect/moderate certainty	Large effect/moderate certainty		Moderate effect/moderate certainty	Large effect/low certainty	?/very low certainty			
CBT vs other		Small effect/moderate certainty			Effective/low certainty **		?/very low certainty	Little or no difference/low certainty	?/very low certainty
TF-CBT vs TAU *						Moderate effect/moderate certainty	Large effect/low certainty		
TF-CBT vs other *						Moderate effect/moderate certainty	Little or no difference/low certainty		
TF-CBT vs EMDR						?/very low certainty	?/very low certainty		
DBT vs TAU				Small effect/low certainty				Moderate effect/low certainty	
Family therapy vs TAU *				?/very low certainty		?/very low certainty	?/very low certainty	Little or no effect/moderate certainty	
Family therapy vs individual/other therapy		Little or no effect/moderate certainty		?/very low certainty	?/very low certainty				?/very low certainty
Psychodynamic vs other				?/very low certainty		?/very low certainty			
EMDR VS TAU						?/very low certainty	?/very low certainty		
Parent training vs TAU	Moderate effect/low certainty					?/very low certainty	?/very low certainty		
Group therapy vs other								Little or no difference/moderate certainty	?/very low certainty
Interpersonal vs TAU				Moderate effect/low certainty				?/very low certainty	
**Other treatments**									
Physical activity vs TAU	Moderate effect/low certainty			Moderate effect/low certainty					
Massage therapy vs TAU	?/very low certainty						?/very low certainty		
Mindfulness vs TAU		Small effect/moderate certainty		Small effect/low certainty					
**Nutrition and supplements**									
Polyunsaturated fatty acids vs placebo	Little or no difference/moderate certainty			?/very low certainty					
**Pharmacological treatments**									
Aripiprazol 10 mg sammenliknet med aripiprazol 30 mg			?/very low certainty						?/very low certainty
Risperidone vs olanzapine			?/very low certainty						?/very low certainty
Psychological vs pharmacological		Small effect (in favour of psychological)/low certainty		Little or no difference/low certainty	Little or no difference/low certainty				
**Combination therapies**									
CBT and pharmacological treatment vs CBT ***	Little or no difference/low certainty	Moderate effect/low certainty		?/very low certainty					
CBT and pharmacological treatment vs pharmacological treatment ****	Large effect/low certainty	?/very low certainty	?/very low certainty						?/very low certainty

* Several outcomes available, outcomes with larger effect and certainty included here.

** Difficult to assess size of effect, review authors report the intervention as “effective”.

*** Several outcomes available, outcomes with larger effect and certainty included here. Pharmacological treatment included stimulants (ADHD), SSRI (depression) and TCA (anxiety).

**** Several outcomes available, outcomes with larger effect and certainty included here. Pharmacological treatment included antipsychotics (psychosis) and SSRI (anxiety). Pharmacological treatment for bipolar and ADHD not specified.

Most treatment evaluations were associated with low or very low certainty. The certainty was higher for treatment evaluations including CBT, which also demonstrated moderate to large effects compared to treatment as usual across different diagnostic groups. DBT compared to treatment as usual has been evaluated for depression and self-harm with low to moderate effect sizes on primary symptoms (low certainty). Physical activity is found to have a moderate effect on primary symptoms of ADHD and depression (low certainty). Mindfulness interventions also result in small but beneficial effects compared to treatment as usual (TAU) for anxiety and depression (moderate to low certainty).

Little or no important difference in effect was found when comparing psychological treatments with pharmacological treatments for depression and OCD, however a small but beneficial effect in favour of psychological treatment was found for anxiety (all low certainty). Combination therapy of CBT and pharmaceutical treatments was found to have a moderate effect on anxiety, but there was little or no difference for ADHD when compared to psychological treatment alone. The certainty of the evidence for combination therapy compared to pharmacological therapy alone, was very low for three out of four diagnostic groups evaluating this treatment comparison (anxiety, bipolar and psychosis). For ADHD, combination treatment was found to produce large effects on the primary outcome, although with low certainty.

Treatment comparisons including psychoeducation (vs TAU), family therapy (vs TAU), psychodynamic (vs TAU), Eye Movement Desensitization and Reprocessing (EMDR) (vs TAU), massage therapy (vs TAU) is very uncertain across all diagnostic groups evaluated.

## Discussion

### Limitations and strengths

Our analysis is based on eight recent overviews of overviews summarising the evidence reported in 116 systematic reviews. ^11^^–^^18^ This analysis includes only direct comparisons of treatments evaluated in high quality systematic reviews. To our knowledge this is the first review of its kind mapping treatments for child and youth mental health, and our findings is of great relevance to clinicians, funders of new research initiatives and researchers providing a blueprint for planning future research activities. Furthermore, acknowledging research uncertainties is an important step-stone in good patient care.

One limitation to our work is that research is accumulating rapidly in this field, and that while this report is being written, new evidence may have emerged. However, considering the substantial evidence gaps we identified we are confident that our conclusions may continue to be valid some time to come. Another limitation may be that our analysis is based on reviews, and that the results are impacted by the methodological choices and reporting by the authors of the individual reviews included in each OoO, but also by the inclusion criteria by the OoO. For example, this analysis does not include placebo-controlled pharmaceutical studies or comparisons of preventive treatments. As such, we would like to emphasise that there is a large body of evidence evaluating these treatment comparisons and that the results of these and the corresponding evidence gaps should be considered together with our analysis. Network analyses of indirect comparisons are an important contribution to this evidence base. ^26^ Furthermore, our analysis is based on treatments evaluated in specific diagnostic groups. Other reviews have summarised the effects of interventions in diverse patient groups (not restricted to diagnostic criteria). Thus, implementation of findings coming out of our analysis should take into consideration the findings of such transdiagnostic reviews.

### Summary of results

We identified 200 treatment comparisons. The most striking finding of this review is the short follow-up times for most treatment comparisons. This is the case for pharmaceutical treatments in particular. Approximately 40% of all treatment comparisons included only short-term follow-up (<3 months), with only 10% of the treatment comparisons including a follow-up beyond 12 months. Thus, there is great uncertainty associated with the long-term effects of mental health treatments for children and young people.

We found large diversity in treatments evaluated. Our descriptive analysis shows that the largest diversity can be found for ADHD and less diversity was found for OCD. Furthermore, from the research that has been conducted so far it can be deducted that the etiology and assumed relevant interventions are understood differently depending on the diagnosis. Some conditions are, for instance, addressed mostly by combination treatments or pharmaceutical treatments alone whereas others are treated with psychological or psychosocial interventions. For example, most treatments for bipolar disorder were pharmacological, whereas treatments for children with PTSD/trauma were all psychological or psychosocial therapies. Treatments for ADHD and psychosis include a combination of both pharmaceutical and psychological or psychosocial treatments. This is an important contribution to the epistemology of mental health, and the understandings of what may reduce or increase mental health depending on the symptoms we identify (diagnosis).

The certainty of most treatment outcomes is low or very low. However, there are treatments with promising effects, which also seem to be effective across diagnostic groups. CBT demonstrates moderate to large effects (compared to treatment as usual) across four diagnostic groups. DBT therapy, physical activity and mindfulness interventions also result in small to moderate beneficial effects compared to TAU (moderate to low certainty). Combination therapies including CBT and pharmacological treatments was either found to have similar effects on primary symptoms compared to either CBT or pharmacological treatment alone, or to be more effective (low certainty).

### Implications for research and practice

Practitioners and decision-makers should be aware of important research uncertainties and make these explicit in communication with patients and when developing guidelines. Our report may be an important platform for doing so.

For many treatment evaluations the evidence is limited. Nevertheless, some treatments have shown convincing treatment effects and should be considered as first choice when treating children and young people. Some treatments identified in our review may be well documented for use in adults and may therefore also be applied to younger people. Although this may be theoretically sound, the transferability to and the effectiveness for younger people is unclear and should be addressed. There is therefore a need for more high-quality research on the effectiveness of treatments with uncertain effects on children and young people. Future research efforts should also include long-term follow up assessments, as this is fundamental to patient safety.

We have made no judgements about the clinical importance of the effect-sizes we mapped in this review, as this is a decision which is best left up to health professionals and their patients. Furthermore, we did not review or make any judgements about which outcomes were evaluated in the treatment comparisons we mapped. For pragmatic reasons, our analysis depended on the primary outcome (change in symptoms) for each diagnostic group using statistical rule of thumb thresholds. It is important to emphasise that any changes in symptomatology should be supplemented with measuring other relevant outcomes. The opinions of patients and health professionals about the relevance of outcomes may differ from those of researchers. Thus, future studies should include the opinions of patients and should consider any established core outcome sets. ^27^^,^^28^

## Conclusions

Our analysis provides essential information through a mapping review about the state of the art of existing evidence.

We identified a wide range of treatments evaluated for use in children and young people, including psychological, social, dietary, physical activity, skills training and pharmacological interventions. Based on this review, it can be observed that some diagnoses are treated mostly with pharmaceutical therapies (such as bipolar disorder) or pharmaceutical or psychological therapy alone or in combination (such as anxiety and psychosis) and others are treated with a broader spectrum of therapeutic approaches (such as depression and attention deficit / hyperactivity disorder).

With few exceptions, the evidence supporting most treatments is of low to very low certainty. CBT demonstrates moderate to large effects across four diagnostic groups with moderate to low certainty. DBT, physical activity and mindfulness interventions also demonstrate small to moderate beneficial effects compared to TAU (moderate to low certainty). Most concerningly, we observed that the majority of the existing treatment evaluations included very short follow-up measurements. Health professionals and policy makers should consider these uncertainties when communicating with patients. Future research efforts should target important research uncertainties identified in this review and plan for longer follow-up times.

## Data availability

### Underlying data

Zenodo: Included comparisons_mapping review_2022. https://doi.org/10.5281/zenodo.6948764. ^29^

This project contains the following underlying data:

- Treatment comparisons mapping review_2022_english.xlsx (comparisons included in the analysis of this paper).

Zenodo: Supporting materials_mapping review_2022. https://doi.org/10.5281/zenodo.6815213. ^30^

This project contains the following underlying data:

- Appendix_mapping_june 2022.docx (systematic reviews included in the OoOs).

Data are available under the terms of the Creative Commons Attribution 4.0 International license (CC-BY 4.0).
