# Supplementary material for: Mental health interventions targeting children and young people: A mapping review of interventions, follow-up, and evidence gaps
Source: F1000Res. 2025 May 8;12:2. Originally published 2023 Jan 3. [Version 2] doi: 10.12688/f1000research.123561.2 (PMC12877472; doi:10.12688/f1000research.123561.2)
Supplement: Peer review history

# Reviewer report: Reviewer response for version 2

**DOI:** 10.5256/f1000research.181260.r449641

**Published:** 2026-02-05

**Authors:** Claire Van Der Westhuizen

**Affiliations:** 1 University of Cape Town, Rondebosch, Western Cape, South Africa

Thank you for the opportunity to review this paper. This is an informative paper, and I believe that the authors have adequately addressed the comments in previous rounds of reviews. The limitations of the review are clearly described. I would be interested to know if the overview of overviews paper included are available in English.

Is the work clearly and accurately presented and does it cite the current literature?

Yes

If applicable, is the statistical analysis and its interpretation appropriate?

Not applicable

Are all the source data underlying the results available to ensure full reproducibility?

Yes

Is the study design appropriate and is the work technically sound?

Yes

Are the conclusions drawn adequately supported by the results?

Yes

Are sufficient details of methods and analysis provided to allow replication by others?

Yes

Reviewer Expertise:

public mental health

I confirm that I have read this submission and believe that I have an appropriate level of expertise to confirm that it is of an acceptable scientific standard.

# Reviewer report: Reviewer response for version 1

**DOI:** 10.5256/f1000research.135677.r367865

**Published:** 2025-03-22

**Authors:** Heather Eileen Menzies Munthe-Kaas

**Affiliations:** 1 Norwegian Institute of Public Health, Oslo, Norway

This is a very interesting and well written article on an important topic. A few small comments from me:

- Terminology – in this manuscript the terms “mental health” mental illness” and “mental disorders” are used. Best to choose one, and consider carefully the term “mental illness” since ADHD at least, is not considered a mental illness, but rather a neurodevelopmental disorder (paragraph 2)
- Consider acknowledging that this group of conditions are not in the same category. For example, ADHD (neurodevelopmental disorder) and anxiety, depression, bipolar disorder (mood disorders) and the reason for grouping them in this paper (which is not problematic in itself, and is probably representative of the literature, but needs acknowledgment of here). The introduction would benefit from one or two additional sentences about why these specific mental health and neurodevelopmental disorders are included (e.g., they are grouped in existing reviews and OoOs).
- Decision making only has a “-“ when it is used as an adjective (e.g., decision-making tools)
- Consider using the term “user” or “client” instead of “patient”
- Define “living evidence summaries”
- Explain why “overviews of systematic reviews” is shortened to “OoOs” (e.g., also referred to as overview of overviews; OoOs). Also can make more clear that the authors have also conducted and published the included OoOs.
- Missing at the end of the introduction a sentence situating this work in the greater picture of work and what gap this study is addressing.

Methods

 Overall this section could use a bit more details and explanation as well as structure to better distinguish from what was done during OoOs and what is done here (and what was carried over from original SRs) perhaps using a flow chart or diagram to show information/data flow?

- A subtitle indicating that you are describing methods for the OoOs and not the mapping review
- Since the most recent of the OoOs is from February 2021, and this is a very active research field, is this mapping review really the best overview of all treatments (and longest follow up)? Can you explain this approach to summarizing and mapping the evidence when there is likely a large chunk of systematic reviews from the last 4-5 years missing here? Could you also write more about how far back the included SRs in the included OoOs go back to?
- Data collection – could you describe sunburst diagrams and what exactly will be presented in them?
- The members of this research team made the OoO GRADE assessments? How were the GRADE assessments taken from the original systematic reviews used?

Results

- Table 5 – the table is likely perfectly accurate, but it does seem strange that all “very low certainty” are related to uncertain effects (assuming “?” means uncertain effects?) and no other effect estimates are “very low certainty” (e.g., it is possible to have very low certainty in an effect estimate that shows a moderate observed effect size). Also , where ** is used to indicate that it is difficult to assess the size of effect but the review authors report the intervention as effective – this is not rated as very low certainty as compared to low certainty?

Is the work clearly and accurately presented and does it cite the current literature?

Partly

If applicable, is the statistical analysis and its interpretation appropriate?

Yes

Are all the source data underlying the results available to ensure full reproducibility?

Yes

Is the study design appropriate and is the work technically sound?

Yes

Are the conclusions drawn adequately supported by the results?

Yes

Are sufficient details of methods and analysis provided to allow replication by others?

Yes

Reviewer Expertise:

stakeholder engagement in research, systematic review and qualitative evidence synthesis methods

I confirm that I have read this submission and believe that I have an appropriate level of expertise to confirm that it is of an acceptable scientific standard.

# Response

**Published:** 2025-04-28

**Authors:** Astrid Dahlgren

**Peer-reviewer 2 Heather Eileen Menzies Munthe-Kaas**

1. Terminology – in this manuscript the terms “mental health” mental illness” and “mental disorders” are used. Best to choose one, and consider carefully the term “mental illness” since ADHD at least, is not considered a mental illness, but rather a neurodevelopmental disorder (paragraph 2) **Author response: **This is a very valid point, and we agree that this should have been more carefully formulated. ADHD is indeed a neurodevelopment disorder of which some people with this condition will need therapy, training or support. We have now used “mental health” and “mental disorder” throughout the manuscript which are terminology that encompass both mental illness and neurodevelopment disorders.
2. Consider acknowledging that this group of conditions are not in the same category. For example, ADHD (neurodevelopmental disorder) and anxiety, depression, bipolar disorder (mood disorders) and the reason for grouping them in this paper (which is not problematic in itself, and is probably representative of the literature, but needs acknowledgment of here). The introduction would benefit from one or two additional sentences about why these specific mental health and neurodevelopmental disorders are included (e.g., they are grouped in existing reviews and OoOs). **Author response: **The diagnostic groups included in this paper are under the common descriptor “mental disorder” and is defined by the World Health Organisation as “a clinically significant disturbance of an individual’s cognition, emotional regulation, or behaviour” https://www.who.int/news-room/fact-sheets/detail/mental-disorders. Although the etiology of the included disorders may be different, they are in many cases associated with distress or impairment in important areas of functioning and are often treated for their symptoms by methodologies with shared epistemology and by the same services and professionals. To make this clearer we have added a sentence to the introduction.
3. Decision making only has a “-“ when it is used as an adjective (e.g., decision-making tools) **Author response:** Thank you, this has been corrected.
4. Consider using the term “user” or “client” instead of “patient” **Author response:** We have changed “patient groups” to diagnostic groups to be more precise and have used “patient” where patient rights or roles are described.
5. Define “living evidence summaries”. **Author response: **A definition has been added to the manuscript. 
6. Explain why “overviews of systematic reviews” is shortened to “OoOs” (e.g., also referred to as overview of overviews; OoOs). Also can make more clear that the authors have also conducted and published the included OoOs. **Author response: **We have now rephased to using overviews of overviews throughout the manuscript. We have stated under the sub-heading: “The evidence: Inclusion criteria and search strategy “ that we authored the OoOs.
7. Missing at the end of the introduction a sentence situating this work in the greater picture of work and what gap this study is addressing. **Author response: **This has now been added.

**Methods:**

1. Overall this section could use a bit more details and explanation as well as structure to better distinguish from what was done during OoOs and what is done here (and what was carried over from original SRs) perhaps using a flow chart or diagram to show information/data flow? **Author response: **The flowchart of data is shown in Figure 1. Preferred Reporting Items for Systematic Reviews and Meta-Analyses (PRISMA) flowchart. We agree that this section needed to be restructured and have now done so to accommodate the peer-reviewers feedback. 
2. Since the most recent of the OoOs is from February 2021, and this is a very active research field, is this mapping review really the best overview of all treatments (and longest follow up)? Can you explain this approach to summarizing and mapping the evidence when there is likely a large chunk of systematic reviews from the last 4-5 years missing here? Could you also write more about how far back the included SRs in the included OoOs go back to? **Author response: **Our mapping review was first published in this journal in January 2023, and as stated in the manuscript, the data collection took place between September 2021 and December 2022. Thus, our report was published very briefly after the data collection was completed. The OoOs we used for our synthesis were considered up-to date at the point of our data collection (these were either new first editions or had been semi-annually or annually updated) at the time. The database from which the OoO include their systematic reviews were updated monthly. To be included, a systematic review should be newer than five years but would not be replaced unless a systematic review considered to be more updated was identified- following the principle of the best available evidence to inform practice. All literature reviews suffer the risk of being outdated once they are published. Judgements about the relevance of the evidence and to what extent new research would change the conclusions is dependent on the research question, the intended use and the robustness of the existing evidence. At the time of the publication of this paper, we were interested in exploring the diversity of treatment interventions for the included mental disorders but also to describe the follow-up times in the existing studies. We also wanted to provide an overview of the effects of these interventions and their associated certainty of the evidence across diagnostic groups. Our conclusion is based on a very large evidence base of 116 systematic reviews including 200 treatment comparisons. Even though we expect new primary studies to have been published from the time our review was first published- we are confident that our conclusions have not been changed substantially:
  1. Some mental illnesses are treated mostly with pharmacological, or psychological therapies or a combination of these. Some are treated with a broader spectrum of therapies. 
  2. The evidence supporting most treatments is of low to very low certainty. 
    Very few treatment evaluations include follow-up assessments beyond 12 months. 
  3. Some treatments are effective across populations, such as cognitive behavioral therapy 
3. Data collection – could you describe sunburst diagrams and what exactly will be presented in them? **Author response: **A sentence has been added to improve clarity. 
4. The members of this research team made the OoO GRADE assessments? How were the GRADE assessments taken from the original systematic reviews used? **Author response: **Yes, one or more of the author team was involved in the OoO published in the evidence portal. We extracted the GRADE-judgements for the primary outcome (in other words- we did not make any further assessments as part of this mapping review considering GRADE). In the methods section we also state that: The primary outcome was conceptualised as overall symptomatology associated with the relevant condition, such as anxiety symptoms for those with anxiety and depression symptoms for those with depression. In cases where the same outcome was assessed using different outcome measures, we extracted the outcome with the longest follow up and highest certainty. If a review did not report findings on overall symptoms, we reported other symptoms as proxy, for example inattention in treatment of ADHD when total symptoms were not available.

**Results**

1. Table 5 – the table is likely perfectly accurate, but it does seem strange that all “very low certainty” are related to uncertain effects (assuming “?” means uncertain effects?) and no other effect estimates are “very low certainty” (e.g., it is possible to have very low certainty in an effect estimate that shows a moderate observed effect size).  Also , where ** is used to indicate that it is difficult to assess the size of effect but the review authors report the intervention as effective – this is not rated as very low certainty as compared to low certainty? ​​​​​​​ **Author response: **The GRADE-framework recommends that outcomes of very low certainty are not reported as the confidence in the findings is too low. In our table this is indicated as “?” (https://www.gradeworkinggroup.org/publications.html). For two of the comparisons, there was uncertainty about the *size* of the effect, but not *direction. *Thus, we were not able to classify the size of effect according to the pre-defined thresholds. These outcomes have been highlighted in the table, both of these were downgraded for precision in the GRADE-assessments and considered to be of low certainty in the original OoOs

# Reviewer report: Reviewer response for version 1

**DOI:** 10.5256/f1000research.135677.r199270

**Published:** 2023-09-06

**Authors:** Hiran Thabrew

**Affiliations:** 1 University of Auckland, Auckland Hospital, Auckland, New Zealand

Thank you for the opportunity to review this interesting paper summarizing results of a mapping review of interventions for common child and adolescent mental health problems.

 The strengths of this paper are:

1. The use of a comprehensive national database of evidence. 
2. The inclusion of most common child and adolescent mental health problems.
3. The clearly presented information about how the study was conceptualized, designed and implemented, including adequate supplementary material with which the review may be replicated.
4. Clear tables and figures to summarize results.
5. Inclusion of length of follow-up data to highlight the short-term nature of most studies.

Areas for improvement are as follows:

1. My main concern about this paper is the limitation of included studies to those which had used an active comparator (not placebo). This is despite the source OoOs including studies with any type of comparator:
*Statement from mapping review: "The OoOs excluded studies where pharmacological interventions were not compared to active interventions, thus placebo-controlled studies were excluded."*
*Statement from protocol of ADHD review: "Comparator(s)/control: Any interventions or treatment as usual (TAU)."*
  I'm not sure why placebo controlled studies were excluded from the mapping review, and as a result, its findings are technically interesting, but of limited clinical benefit. A key example is how methlyphenidate pharmacotherapy for ADHD is not explicitly mentioned in Table 5, even though it is the mainstay of treatment for this condition worldwide. Greater explanation of this issue (or, dare I ask for it, a reworking of the analysis including all types of studies) would be useful.
2. Using colors more consistently in the sunburst diagrams (Figures 2-10) to make it easier for the reader to compare the weight of evidence for each type of therapy across different conditions (e.g., medication is blue in one diagram, yellow in another).
3. Please explain the colors and question marks used in Table 5.

Is the work clearly and accurately presented and does it cite the current literature?

Yes

If applicable, is the statistical analysis and its interpretation appropriate?

Yes

Are all the source data underlying the results available to ensure full reproducibility?

Yes

Is the study design appropriate and is the work technically sound?

Partly

Are the conclusions drawn adequately supported by the results?

Yes

Are sufficient details of methods and analysis provided to allow replication by others?

Yes

Reviewer Expertise:

Child and adolescent mental health clinician and researcher

I confirm that I have read this submission and believe that I have an appropriate level of expertise to confirm that it is of an acceptable scientific standard, however I have significant reservations, as outlined above.

# Response

**Published:** 2025-04-28

**Authors:** Astrid Dahlgren

Peer-reviewer .1 Hiran Thabrew

1. My main concern about this paper is the limitation of included studies to those which had used an active comparator (not placebo). This is despite the source OoOs including studies with any type of comparator:

**Author response:** This limitation was only for pharmaceutical therapy compared with placebo- not other mental health interventions. The reason for this was that the evidence base for this review was a series of overviews of systematic reviews (OoO) conducted as part of the development of a national evidence portal commissioned by the Norwegian directorate of health to inform clinical practice in Norway as part of the launching of Clinical Pathways mental health for children and young people. In this context the relevance of placebo-controlled pharmaceutical studies was judged by policymakers and mental health practitioners to be low as pharmaceutical treatment are only advised to be considered when other treatments are found not to be effective. Therefore, the focus was on pharmaceutical treatments compared to other active treatments used alone or in combination with other treatments.

 As the peer-reviewer rightly states, the OoOs  searched for and included placebo-controlled comparisons for pharmaceutical therapies, but the findings of these comparisons were only reported descriptively as supplemental material in the OoOs without full data extraction and GRADE-assessments.

 In regard to the peer- reviewer’s comments about Methylphenidate, we agree that this is a treatment of relevance to clinical practice and is also one of the most commonly used pharmaceutical treatments in Norway for young people under 18 with ADHD.

 Methylphenidate is included in the review as 1 of 57 unique treatments for ADHD in figure 2: Unique intervention categories evaluated for attention deficit / hyperactivity disorder (ADHD).

 Table 5 reports treatment comparisons that have been tested for more than 1 diagnosis and on a higher aggregated level as evaluated in the systematic reviews (in this case “stimulants” or in the broader term “pharmacological treatment”). Where type of pharmaceutical treatment is reported in the systematic reviews this has been described by us in the notes to table 5. See for example under “Combination therapies”. We hope this helps to clarify the peer-reviewers concerns.

 2. Please explain the colors and question marks used in Table 5.

**Author response:** Explanation for colour-coding is as follows: Yellow is used for low certainty, red for very low certainty and green for moderate certainty.  A sentence has been added to the manuscript.
